# Patient education interventions for adolescent and young adult kidney transplant recipients- a scoping review

**DOI:** 10.1371/journal.pone.0288807

**Published:** 2023-07-17

**Authors:** Michael Corr, Clare McKeaveney, Fina Wurm, Aisling Courtney, Helen Noble

**Affiliations:** 1 School of Medicine- Queen’s University Belfast, Belfast, Northern Ireland; 2 School of Nusring- Queen’s University Belfast, Belfast, Northern Ireland; 3 Regional Nephrology & Transplant Unit-Belfast Health and Social Care Trust, Belfast, Northern Ireland; Monash University, AUSTRALIA

## Abstract

**Background:**

Adolescence and young adulthood are high risk periods for kidney transplant recipients. The reasons for this are complex; but are predominantly thought to be due to poor adherence to immunosuppressive medications. Patient education can help support young recipients to reduce their risk of behaviour-related transplant loss. The aim of this review was to understand what is known about education interventions targeted at adolescent and young adult kidney transplant recipients.

**Methods:**

Systematic scoping review methodology was utilised. Six online databases were searched for suitable articles. Articles were selected for full text review following title and abstract screening. Articles deemed eligible to be included in the review had data extracted, which were qualitatively analysed using thematic analysis. Findings were validated through a consultation exercise with both young recipients and healthcare professionals.

**Results:**

29 studies were eligible for inclusion in the review. There was a high level of heterogeneity in the content, mode, design, and measurement of efficacy of interventions in the selected studies. Traditional face-to-face education and transition clinics were the most common educational interventions. Using technology to enhance patient education was also a major theme identified. Few studies reported using educational theory or involving patients in intervention design.

**Discussion:**

Four key research gaps were identified. 1.) Lack of educational theory in intervention design 2.) Lack of patient/ stakeholder involvement 3.) Identifying best way to measure efficacy 4.) identifying novel future research questions within already well established paediatric and educational frameworks. Addressing these gaps in future research will help inform best-practice in this vulnerable population.

## Background

Adolescence and young adulthood represent a high-risk period for kidney transplant recipients [1]. Potential increased immunological activity combined with suboptimal adherence to important immunosuppressive medications puts young recipients at increased risk of premature graft loss [2,3]. Post-transplantation adolescents and young adults enjoy the best short-term outcomes of any age cohort; however, they have the highest rates of graft loss in the long-term [4]. The reported median survival of a deceased donor kidney in the whole population is 10 years vs. 7 years in young adults [5].

The challenges following premature transplant loss in this age group are profound. The return to dialysis is associated with high morbidity and mortality [6]. Re-transplantation is often complicated by the development of Human Leukocyte Antigen antibodies meaning young people may face many years on dialysis before a suitable kidney is available [7]. All this occurs in the context of the young person’s life which is rapidly changing where dialysis can severely disrupt key life events such as, increasing independence from parents, ongoing education, entering work, development of non-familiar relationships and starting families of their own [8,9].

Prevention of premature graft loss in adolescent and young adult transplant recipients can be challenging. Interventions are usually focussed on patient education and self-management given their reported poor adherence to medications and high risk of being lost to follow-up [10]. Patient education initiatives are often complicated by high rates of mental health conditions in young transplant recipients potentially exasperated by the COVID-19 pandemic as seen in others with chronic health conditions [11,12]. Often those that require the most assistance psychologically are the same individuals who find it most difficult to engage with services [13]. Further challenges arise due to the impact that end-stage kidney disease (ESKD) has on neurocognitive development of young people and learning disabilities associated with syndromes linked to the development of ESKD at a young age [14]. Healthcare professionals, often seen as authoritative figures, can find themselves central to a young person’s rebellion hindering meaningful interaction [15]. This can be further complicated when care has been transitioned from paediatric to adult services where relationships between recipient and staff are new. Transition has long been associated with increased risk of adverse events in both transplant recipients and other chronic diseases [16].

Given the challenges; those tasked with assisting adolescent and young adult transplant recipients have radically changed their clinics and education programmes. Many services now offer specific clinics for adolescents and young adults with an enhanced multidisciplinary team including social and youth workers [17]. Others have used the opportunities technology has provided to engage with young recipients [18]. In the United Kingdom, the transition process has been overhauled with formal transition programmes such as, “Ready, Steady, Go” to prepare young people moving from paediatric to adult care [19]. There has been an increase in research reporting patient education interventions and a new focus on patient centred studies and outcomes through initiatives such as SONG-kids [20].

The aim of this scoping review is to systematically map the literature related to any form of patient education interventions for adolescent and young adult kidney transplant recipients. This will provide a comprehensive summary of the varying patient education interventions that have been reported whilst also allowing a critical reflection on remaining challenges and gaps yet unanswered in the existing research.

## Methods

A scoping review methodology was selected given the expected heterogeneity of patient education interventions. A scoping review is a form of knowledge synthesis which maps out key concepts, types of evidence and gaps in a research field in a systematic manner. This study was completed adhering to the methodological recommendations and steps as laid out by Colquhoun et al., 2014, Levac et al., 2010, Arksey, 2005 [21–23]. Reporting of the review was completed in conjunction with the guidance of PRSIMA extension for reporting scoping reviews [24]. The completed PRISMA checklist can be viewed in the S1 Table.

### Eligibility criteria

All primary research reporting patient education interventions for adolescent (defined as aged 11–18 by World Health Organization) and/or young adult (defined as aged 18–24 by World Health Organisation) kidney transplant recipients were eligible for inclusion. For the purposes of this review education intervention was defined as a change in practice or novel method in delivering patient education. All study designs were included as eligible; professional reports and editorials were not included. Studies which included other solid organ transplant recipients were only included if the data pertaining to only kidney transplant recipients could be separated.

### Search strategy

A search strategy was designed with the assistance of a medical librarian. Six online databases were searched for relevant articles- OVID-Medline, EMBASE, Web of Science, Scopus, CINAHL, PsycInfo from inception until 15^th^ December 2022. A targeted grey literature search was also conducted. An example search strategy can be viewed in the S2 Table.

### Study selection and data extraction

Following the online search of databases titles and abstracts were reviewed for appropriateness for full-text review against the inclusion criteria by two reviewers. Full text review was conducted independently by two researchers. Where there was disagreement regarding inclusion or exclusion of a study consensus was reached by the two researchers. Where consensus was not reached arbitration was achieved via a third-party member of the study team.

Data were then extracted from each included article using a pre-designed data extraction tool. Where a review article was found references were reviewed for possible inclusion in the study. A completed extraction tool can be found in the S3 Table.

### Data analysis

A mixed methods approach was taken for data synthesis as data identified was both quantitative and qualitative in nature. The mixed methods approach described by the Joanna Briggs Institute for mixed method data analysis in synthesis reviews was adopted [25]. Convergent integration of the data was applied, i.e., quantitative data was extracted and transformed into textual descriptions to create qualitative data for analysis. This allowed the newly formed qualitative data from quantitative studies and the data from qualitative studies to be coded using traditional content thematic analysis [26]. Scoping review methodology assessment of study quality or for potential biases was not conducted as per guidelines.

Results of the thematic analysis were validated via a consultative exercise with young transplant recipients and healthcare professionals involved in transplant education currently as prescribed by the scoping review methodology. Key findings from the scoping review were presented to the group and reflections of their own experiences and challenges in transplant education were discussed to assess thematic validity.

## Results

**Fig 1:**
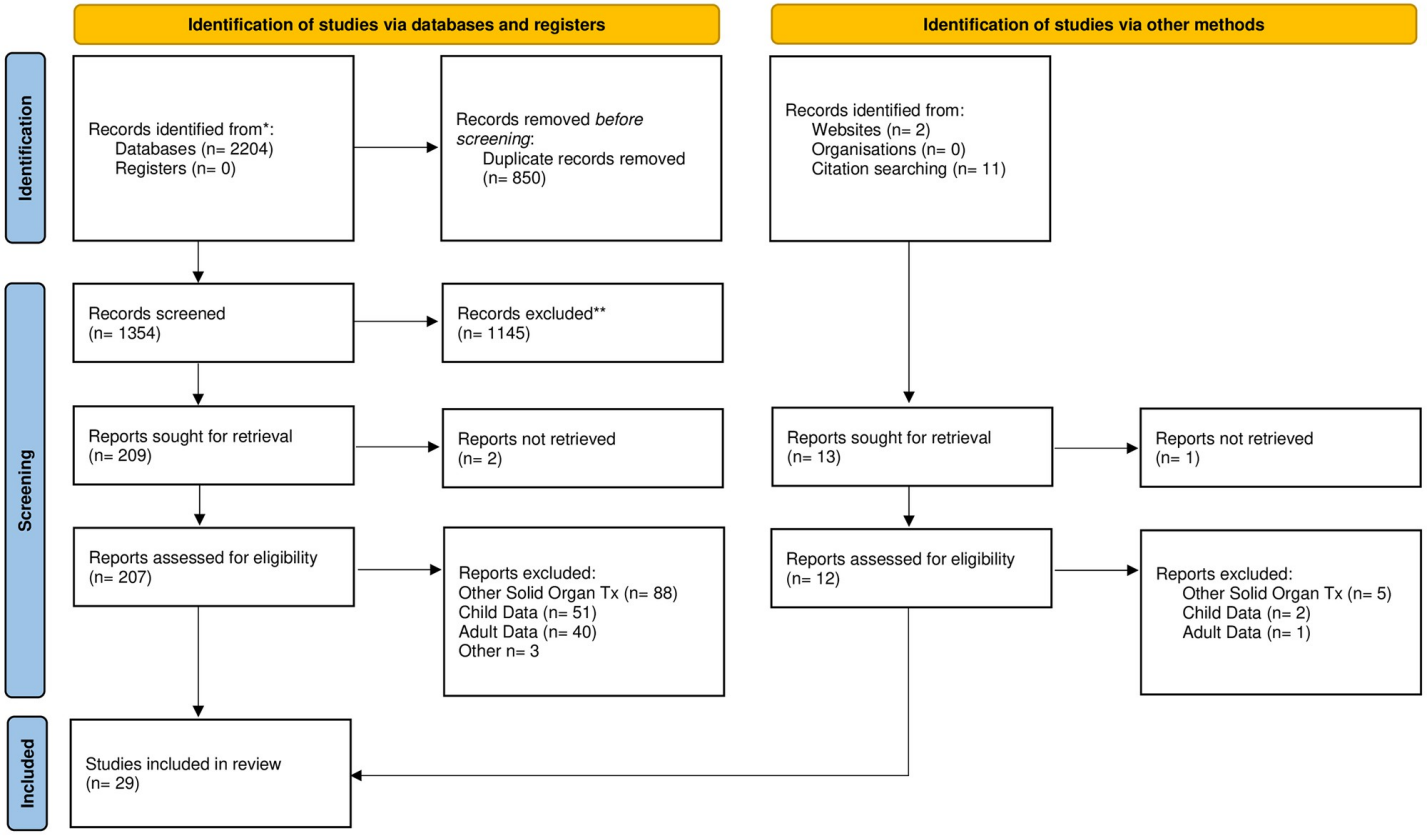
PRISMA flow chart. *From*: Page MJ, McKenzie JE, Bossuyt PM, Boutron I, Hoffmann TC, Mulrow CD, et al. The PRISMA 2020 statement: an updated guideline for reporting systematic reviews. BMJ 2021; 372:n71 doi: 10.1136/bmi.n71. For more information, visit: http://www.prisma-statement.org/.

A total of 29 articles were eligible for inclusion in this scoping review. A PRISMA flow chart demonstrating stages of article selection can be found below (Fig 1) [27].

### Study characteristics

Of the 29 articles included 14 articles had both adolescent and young adult participants [28–41], 13 were studies that only included adolescents [42–54] and only two studies exclusively recruited young adults [55,56]. Most of the studies were based in North America (17 studies) followed by Europe (10 studies) and a single study from both Israel [34] and Australia [36]. There were a range of both qualitative and quantitative methodologies utilised. Five studies were qualitative based [34,36,38,39,45], 3 studies used a mixed-methods approach [47,50,56] whilst the rest were quantitative in design. There were four randomised-controlled trials [29,33,46,49]. Most of the studies were small single centre studies that were observational post-intervention or cross-sectional in design. Several studies were retrospective observations years after the intervention to assess impact on clinical outcomes [32,42,48,51,54,55].

### Content of education delivered

The primary focus of most studies was increasing adolescent and young adults’ health knowledge of their condition. Interventions aimed to develop understanding and hence self-management skills of young recipients and assist them in taking more responsibility for their care [35,46,55]. Many of the education interventions were focussed on increased knowledge of medications taken by the recipients, including their role in managing their transplant as well as potential complications and side effects [30,32]. Two studies aimed to increase emotional wellbeing, educate on mental health issues, or assist young recipients to develop coping mechanisms/ resilience [39,41]. Two studies aimed to educate and encourage young recipients to increase their physical activity and improve cardiovascular health [28,53]. A single study aimed to educate young recipients about appropriate hydration and water intake [49]. There was one study that aimed to address sun-health education given the high risk of skin cancers in this cohort [29], whilst another study investigated contraceptive/ reproductive health education female transplant recipients received [40].

### Education mode

A range of modes of education were used for adolescent and young adult transplant recipients, with some studies using a combination of approaches. Most of the education was delivered in a traditional face-to-face format where either the researcher or a member of the healthcare team gave education directly to the patient and/or their family [35,55]. The training or specific role of those providing the education was often poorly described however within healthcare teams members of nursing staff most often provided the education [29,35]. Some studies used small groups to deliver education to recipients with in-built peer-support and education [36] or emotional wellbeing used therapy/counselling sessions [43]. Ten studies in total used technology as part of their interventions. Technological modes for education delivery ranged from online-based education programmes/resources to the use of devices including pedometers, video games, electronic water bottles and electronic pill boxes to promote health behaviours [28–30,47,48,53]. Very few studies investigated outcome differences related to mode of education, indeed many of the technology-based studies reported were feasibility/acceptability studies. Bottcher et al. did compare different modes of education and found an integrated model combining both online learning with face-to-face sessions was more effective than online sessions alone [29].

### Educational interventions models and designs

Most articles in this review did not provide any information on educational design, theoretical models or guiding principles on which programs were based. One study utilised the teach-back method to assess what the patient had learnt and to identify ongoing knowledge gaps [30]. One further study used, “Action-Focused Problem-Solving,” methodology as a framework for identifying and solving problems related to adherence [32,33]. Studies related to adherence of medications often used the health belief model to understand potential barriers to adherence and based their interventions on this understanding [45]. The amount (6) of studies that specifically outlined stakeholder exercises or involvement of patients in co-design of their interventions was limited [32,33,36,38,46,47].

There was a wide heterogeneity in the amount of information provided by individual studies regarding their interventions. Most only briefly mentioned their interventions, whilst some authors fully outlined and described complex interventions like transition clinics, content of e-learning or workshops; helping to contextualise their findings and improve reproducibility [33,47]. Transition clinics were the most common intervention described in the studies though these ranged in the type and delivery of education delivered; some were primarily health focussed whilst others included social/youth workers who covered a range of topics from relationships, career goals and sexual health [30,35,36]. E-learning through web-based platforms was the second most studied intervention [29,35,44], though these were often linked with further interventions such as coaching sessions. Full details on each individual study’s intervention can be found in the S3 Table.

### Outcomes measured and their effectiveness

Three studies focussed on educational-based outcomes: ranging from validated knowledge assessments taken pre and post intervention and questionnaires/interviews assessing participants confidence in individual topics [29,30]. Few studies included educational outcomes as part of their analysis, however this was not their primary aim [36]. Ten studies investigated outcomes related to psychological wellness, whether through diagnosis of mental health conditions, validated psychometric testing or qualitative interviews to establish if emotional well-being was improved [36,39,41]. Most studies investigated quantitative clinical outcomes such as rate of graft loss, improvement in physiological markers, medication adherence or rates of rejection in their populations [28,47,55].

All studies included in the review reported positive outcomes for their interventions except two which reported no difference between intervention and non-intervention groups [47,48]. The individual quality of each study was not assessed in this review given the scoping review methodology; however, it is important to note the relatively small numbers of participants included in most studies (range of participants 4–440, median number of participant 32). Furthermore, often interventions such as transition clinics were complex and multifaceted in nature making it difficult to assess which component was attributable to the effect observed [35,48,55].

## Discussion

This systematic scoping review demonstrates the educational interventions studied within the literature for adolescent and young adult kidney transplant recipients. Despite a small number of studies reporting interventions there is huge diversity in mode of education, content, design, and outcomes measured. Whilst most studies demonstrated benefits to their intervention, they were often with limited numbers of participants from a single centre.

The most notable knowledge gap identified is the minimal focus on educational theory in the design of interventions and ensuring emphasis high quality education was provided. Few studies involved evidence-based education methods or learning theories [30,32,33]. Few appeared to engage patients with active learning, which has been proven to help learners gain a deeper understanding of content [57]. Most studies failed to include details of how educational information or resources were quality checked or designed with best-practice guidance [58]. Perhaps, given some of the interventions involved e.g., transition clinics, researchers did not consider these as educational interventions. And whilst true that transition clinics have many roles (follow-up, communication between different clinical teams); education and engaging patients is a central component that should be carefully considered in their design [19].

Of note, few studies involved young transplant recipients in the design of their interventions. Limited studies included stakeholder exercises with an aim to understand participants’ educational requirements. Such studies benefited by gaining a deeper understanding of the issues faced by patients and were able to demonstrate how they tailored interventions to meet their needs [32,46]. In recent years there has been an increased focus on including the patient’s voice in trial design and working groups such as SONG-Kids have assisted in helping researchers identify key priorities for young people [20]. There was a predominance of studies from North America and Europe included in this review. There was a marked paucity of research in developing countries and in populations known to require additional patient education support e.g., lower socioeconomic groups and children of immigrants [59]. Within patient education there is an awareness that sociocultural factors can have a significant impact on the effectiveness of interventions [59]. Hence, novel tools described in the literature may translate poorly to other sociocultural and healthcare settings. This should be carefully considered and again, where active involvement of patients in local service design can increase the chance of success. How technology could be used to overcome barriers to patient education in underserved populations (lower socioeconomic classes, populations with less healthcare infrastructure) could be another fruitful area for future study.

Given the clinical challenges in managing young kidney transplant recipients it is perhaps unsurprising researchers were keen to measure effectiveness by clinical outcomes such as graft function and rates of rejection episodes [55]. This however presents challenges, such as, low patient numbers, low clinical event rates (rejection) and the need for long periods of observation following intervention. Given the issues raised above it may be prudent for future research to focus on educational quality and education related outcomes primarily before assessing for clinical related efficacy. Further to this, future work should be guided from previous research in both healthcare and education fields. Transition clinics are well established practice with a significant evidence base supporting their use [19], as has the use of web-based learning in a variety of both patient education and general education research [60]. Creating, well designed need-specific educational interventions for young transplant recipients and identifying best practice within these established frameworks (transition, online education) is of crucial importance. Inclusion of researchers with backgrounds in educational theory and methodology in future study designs may be particularly beneficial.

This review is limited by the fact that English-only manuscripts were included however this did not appear to cause the exclusion of any articles during the selection process. As with the scoping review methodology the individual quality of each study was not assessed. This limits the ability of this review to comment on overall effectiveness of individual interventions. However, it does highlight potential limitations of some of the studies included and where methodological gaps exist. A recent Cochrane review of interventions to address adherence to immunosuppressants in all solid organ transplant recipients (adult and paediatric) concluded that overall, the methodological quality of studies was often poor with high heterogeneity in interventions studied which limited the ability to best inform practice [61].

### Conclusions

Patient education is paramount to reduce adverse incidents in adolescent and young adult kidney transplant recipients. This scoping review has systematically outlined the current literature on patient education interventions for this population. It highlights knowledge gaps such as 1.) Lack educational theory and quality control 2.) lack of patient involvement in study design 3.) careful consideration of how to best measure intervention efficacy 4.) identifying novel future research questions within already well established paediatric and educational frameworks. Addressing these knowledge gaps with future research may help to inform clinicians on how to best design educational programmes for this vulnerable population.

## Supporting information

S1 Table PRISMA checklist. (DOCX)

S2 Table Example search strategy. (DOCX)

S3 Table Data collection tool. (DOC)
