# Supplementary material for: Patient education interventions for adolescent and young adult kidney transplant recipients- a scoping review
Source: PLoS One. 2023 Jul 17;18(7):e0288807. doi: 10.1371/journal.pone.0288807 (PMC10351733; doi:10.1371/journal.pone.0288807)
Supplement: S2 Table — (DOCX) [file pone.0288807.s002.docx]

MEDLINE and EMBASE

| 1 | Kidney transplantation/ or kidney transplant.mp |
| --- | --- |
| 2 | Kidney transplantation/ or renal transplant.mp |
| 3 | 1 or 2 |
| 4 | Adolescent/ or young people.mo. or Young Adult/ |
| 5 | Health education.mp. or Health Education/ |
| 6 | Intervention.mp or early medical intervention/ or internet-based intervention |
| 7 | Health promotion mp. Or Health promotion/ |
| 8 | Behaviour therapy/ or behavioural intervention mp. Or behavioural therapy.mp |
| 9 | transition.mp. or Health Transition/ or Transition to Adult Care/ |
| 10 | 5 or 6 or 7 or 8 or 9 |
| 11 | 3 and 4 and 10 |
