# Supplementary material for: Patient education interventions for adolescent and young adult kidney transplant recipients- a scoping review
Source: PLoS One. 2023 Jul 17;18(7):e0288807. doi: 10.1371/journal.pone.0288807 (PMC10351733; doi:10.1371/journal.pone.0288807)
Supplement: S3 Table — (DOC) [file pone.0288807.s003.doc]

**DATA EXTRACTION TOOL- EDUCATIONAL INTERVENTIONS**

| Authors (reference) | Study Location (Country) | Aim of Study | Number of participants | Adolescents (A) or young adults (YA) | Study Design | Description of intervention (including time period) Mode of delivery | Method of data collection | Methodology of data assessment (framework) | Key Findings | Self-reported Limitations |
| --- | --- | --- | --- | --- | --- | --- | --- | --- | --- | --- |
| Akber A, Portale AA, Johansen KL. Use of pedometers to increase physical activity among children and adolescents with chronic kidney disease. Pediatr Nephrol. 2014 Aug;29(8):1395-402. doi: 10.1007/s00467-014-2787-6. Epub 2014 Mar 20. PMID: 24648129; PMCID: PMC4074553. | USA | Did pedometers increase the physical activity of adolescents with CKD/ ESKD (including transplant recipients). | 25 | Both | Pre-post study | Given a pedometer with education on exercise and reported step count 12 weeks. Technology mode used. | Pediatric Quality of Life Inventory (PedsQL) and six-minute walk (6MW) were administered at baseline and after 12 weeks. | Descriptive statistics | Mean daily step count did not change significantly (+48, 95% CI −48 to +145 steps/day per week). Transplant recipients and patients with CKD increased their activity by 100 steps/day (95% CI −14 to 208) and 73 steps/day (95% CI −115 to 262) each week. Change in physical activity was associated with change in 6MW distance | Self-reported step counts. Occurred during weather seasonal changes so can’t rule out if that was cause of raised step count. |
| Annunziato RA, Parbhakar M, Kapoor K, et al. Can Transition to Adult Care for Transplant Recipients Be Improved by Intensified Services While Patients are Still in Pediatrics? *Progress in Transplantation*. 2015;25(3):236-242. doi:[10.7182/pit2015599](https://doi.org/10.7182/pit2015599) | USA | To determine if a transition coordina-  ator improve outcomes in patients moving from paediatric to adult services. | 22 | A | Retrospective cohort observational trial. | 12 recipients received intensified preparation from social worker. 10 patients received standard care. Took place over 4 years. Face-to-face | Chart analysis | Medicine adherence using Tacrolimus levels. Established SD >2.5= worse outcomes and evidence on non-adherence | Adherence worsened from 1 year before to 1 year after transfer. No benefit demonstrated from increased input. | Small study. Analysis didn’t include measures for confounding results such as socioeconomic class. |
| Bamford J, Wirz L. Piloting Psychology Annual Reviews as a Method of Measuring Psychological Distress and Quality of Life in Paediatric Renal Transplant Patients. Biomed Res Int. 2016;2016:1685362. doi: 10.1155/2016/1685362. Epub 2016 Nov 14. PMID: 27965973; PMCID: PMC5124635. | UK | To assess whether annual reviews with psychologist facilitated identification of patients experiencing psychosocial difficulties. | 120 | A | Prospective observational study, | Yearly appointment with psychologist. All data from one year (2014) only. Face-to-face. | Psychometric measures and final interview. | Measured outcomes. | Identified patients with psychosocial difficulties and helped refer to appropriate services, | Small study, no control. |
| Böttcher S, Buck C, Zeeb H, Laschewski G, Hauer C, Wagner G, Sachse MM. Randomised controlled trial to evaluate the influence of mHealth and eHealth skin cancer prevention education among young organ transplant recipients: the HIPPOlino intervention study. BMJ Open. 2019 Dec 16;9(12):e028842. doi: 10.1136/bmjopen-2018-028842. PMID: 31848158; PMCID: PMC6937057. | Germany, Netherlands and Austria | Whether a multicomponent intervention related to sun exposure was superior to elearning in young transplant recipients | 137 | Both | Randomised controlled trial with one preintervention baseline survey and three follow-up surveys after 6 weeks, 6 and 12 months | Group 1: personal training & personalised messages.  Group 2: elearning alone  Group 3: Online training after one year & regular letters. Over one year.  Mixed Modality. | Self-administered questionnaires validated by cognitive interviews. | Longitudinal difference models | Multicomponent strategy increased patient knowledge and protective behaviours with regards to sun health. | Small study. Behavioural attitudes may have been affected by recall bias, social desirability and issues with self reporting. |
| Chandar JJ, Ludwig DA, Aguirre J, Mattiazzi A, Bielecka M, Defreitas M, Delamater AM. Assessing the link between modified 'Teach Back' method and improvement in knowledge of the medical regimen among youth with kidney transplants: The application of digital media. Patient Educ Couns. 2019 May;102(5):1035-1039. doi: 10.1016/j.pec.2018.12.007. Epub 2018 Dec 12. PMID: 30622001. | USA | Pilot study into whether a “teach back,” IT educational programme increased health literacy amongst young recipients | 16 | Both | Pre and post quasi-experimental trial | Computer based programme with health care worked. To develop a knowledge base about their own condition. Over three months.  Virtual/online based | Binomial sign test at baseline and 3 months | Observed improvement of knowledge | 12 of 16 patients increased their knowledge. | Small study. No control. |
| Dabirzadeh A, Dahhou M, Zhang X, Sapir-Pichhadze R, Cardinal H, White M, Johnston O, Blydt-Hansen TD, Tibbles LA, Hamiwka L, Urschel S, Birk P, Bissonnette J, Matsuda-Abedini M, Harrison J, Schiff J, Phan V, De Geest S, Allen U, Mital S, Foster BJ. Care processes and structures associated with higher medication adherence in adolescent and young adult transplant recipients. Pediatr Transplant. 2021 Dec;25(8):e14106. doi: 10.1111/petr.14106. Epub 2021 Aug 2. PMID: 34339090. | Canada | To identify care processes and structures that were independently linked with better adherence. | 184 | Both | Prospective observational study | Observing for care structures which affect adherence. Face-to-face. | Observations of clinical activity | Adherence measured with BAASIS self-reported tool. Multivariate mixed effects logistic regression models with program as random effect | 2 program-level factors as independently associated with better adherence: minimum number of prescribed blood draws per year for those >3 years post-transplant (per 1 additional) (OR 1.12 [95% CI 1.00, 1.26]; p = .047), and average time nurses spend with patients in clinic (per 5 additional minutes) (OR 1.15 [1.03, 1.29]; p = .017). | Factors measured were identified a priori from authors hypotheses. |
| Foster BJ, Pai ALH, Zelikovsky N, Amaral S, Bell L, Dharnidharka VR, Hebert D, Holly C, Knauper B, Matsell D, Phan V, Rogers R, Smith JM, Zhao H, Furth SL. A Randomized Trial of a Multicomponent Intervention to Promote Medication Adherence: The Teen Adherence in Kidney Transplant Effectiveness of Intervention Trial (TAKE-IT). Am J Kidney Dis. 2018 Jul;72(1):30-41. doi: 10.1053/j.ajkd.2017.12.012. Epub 2018 Mar 27. Erratum in: Am J Kidney Dis. 2019 Apr;73(4):578. PMID: 29602631; PMCID: PMC6019162. | Canada/USA | Whether a novel, multicomponent adherence-promoting intervention, compared with an attention control condition improved medication adherence. | 169 | Both | Unblinded, parallel arm randomized trial to assess the efficacy of a clinic-based adherence-promoting intervention. | Self management strategy and coping. Over 15 months. Mixed modality. | Electronic pill box monitored time and % adherence to medication. Tacrolimus levels also measured. | Intention to treat analysis. Unadjusted ordinal logistic regression. Sensitivity analysis. | Those randomised to intervention group had higher adherence. | Lack of electronic data for some patients may have bias. No evidence of improved clinical outcome. |
| Foster BJ, Platt RW, Dahhou M, Zhang X, Bell LE, Hanley JA. The impact of age at transfer from pediatric to adult-oriented care on renal allograft survival. Pediatr Transplant. 2011 Nov;15(7):750-9. doi: 10.1111/j.1399-3046.2011.01567.x. Epub 2011 | USA | Difference in graft failure in those who transition to adult services early versus delayed transfer. | 440 | Both | Retrospective cross-sectional | Over 4 years. Early vs late transition to adult services. Face-to-face. | Age at transfer to adult services- graft function. | Time-dependent cox models | Those who transferred to adult services early had a 58% increased risk of graft failure. | Retrospective. Based on coding. |
| Freier C, Oldhafer M, Offner G, Dorfman S, Kugler C. Impact of computer-based patient education on illness-specific knowledge and renal function in adolescents after renal transplantation. PediatrTransplantation 2010: 14: 596–602 | Germany | examine the effect of an interactive, computer-based patient educational programme on resulting renal function parameters in adolescent renal transplant recipients | 50 | A | Experimental design | Elearning on medications and elearning behavioural development courses.  Virtual modality. | 9-iQ questionnaire prior to commencement.  Measurement of clinical factors and graft function, immunosuppression levels. | Results and comparison with univariate and multivariate analyses. | Improved knowledge and behavioural knowledge. | Small and not randomised. Failed to demonstrate any change in renal outcomes. |
| Gabay Gillie G, Tarabeih M. "A Bridge Over Troubled Water": Nurses' Leadership in Establishing Young Adults' Trust Upon the Transition to Adult Renal-Care - A Dual-Perspective Qualitative Study. J Pediatr Nurs. 2020 Jul-Aug;53:e41-e48. doi: 10.1016/j.pedn.2020.02.004. Epub 2020 Mar 3. PMID: 32139233. | Israel | What are the barriers to young adults developing relationships with nurses when transitioning care to adult services. How do nurses overcome this? | 22 | Both | Dual perspective qualitative study- interviews, | Transition of care from child to adulthood services. Face-to-face. | In-depth narrative interviews. | Thematic analysis guided by Meleis's framework of effective transitions | Transition a negative experience.  Nusing staff focussing on relationship rather that task orientation helped patients feel more safe, more prepared and more effective. | Small study, single country. |
| Harden PN, Walsh G, Bandler N, Bradley S, Lonsdale D, Taylor J, Marks SD. Bridging the gap: an integrated paediatric to adult clinical service for young adults with kidney failure. BMJ. 2012 Jun 1;344:e3718. doi: 10.1136/bmj.e3718. PMID: 22661725. | UK | If a specialised transition clinic reduced graft rejection | 21 | Both | Before and after study. QIP | Integrated transition clinic with community clinics and workshops. Face-to-face. | Comparison made between two study groups- those directly transitioned into adult and new service | QIP | Six transplants lost in group 1 versus none in interventional group | Small study, not randomised. |
| Ingerski L, Perrazo L, Goebel J, Pai AL. Family strategies for achieving medication adherence in pediatric kidney transplantation. Nurs Res. 2011 May-Jun;60(3):190-6. doi: 10.1097/NNR.0b013e318215fdfe. PMID: 21522031. | USA | To explore patient and family strategies to increase adherence to medication | 17 adolescents and 17 carers | A | Qualitative | Family strategies. Face-to-face. | Semi structured interviews | Thematic analysis | Making it part of the routine (88.2%), verbal reminders* by caregiver (82.4%), caregiver verifying medication was taken* (76.5%), placing medication in a convenient location (76.5%), and using a pillbox (70.6%). | Small study |
| Jose K, Le Roux A, Jeffs L, Jose M. Evaluation of a young adult renal and transplant transition clinic in a regional setting: Supporting young adults and parents' transition to self-management. Aust J Rural Health. 2021 Feb;29(1):83-91. doi: 10.1111/ajr.12683. Epub 2021 Jan 16. PMID: 33452848. | Australia | Evaluate impact of a transition clinic on young people with kidney transplants | 4 transplant patients | Both | Qualitative Study | Transition clinic. Face-to-face. | Semi-structured interviews | Thematic analysis | Four key themes were identified as follows: The Model of Care; Peer support; Transition towards self-management: Building life skills; Suggestions for improvement and limitations of the service model. The non-institutional, informal clinic setting and social/educational activities facilitated engagement, self-management and peer support for young people and parents. Suggestions for improvement included involvement of older peers, additional life skills sessions and a youth worker | Small sample size |
| Kocjančič D, Battelino N, Oblak M, Novljan G. Psychosocial impact of structured transfer of adolescents with kidney transplants to adult services: An interventional national cohort study. Pediatr Transplant. 2022 Dec;26(8):e14373. doi: 10.1111/petr.14373. Epub 2022 Aug 24. PMID: 36000468. | Slovenia | Whether a structured transition protocol improved psychosocial outcomes | 10 | Both | Interventional cohort study | Transition protocol including assessment of psychosocial wellbeing and preparedness for transfer. Face-to-face. | Centre specific questionnaire- mixture of open ended and closed ended questions | Descriptive statistical methods were used to compare pre and post transition intervention | The most frequent concerns regarding upcoming health care were worse accessibility (50%), less supportive and less committed healthcare providers (40%), and deterioration of medical condition (10.0%). After the completed protocol-guided transfer, the patients declared to have no further concerns or worries. Before transfer, 28.9% of the patients' responses rated the amount and relevance of received information and counseling as "Adequate" or "Very adequate," whereas, after the transfer, the proportion of positive responses increased to 48.9%. Anxiety and withdrawn depressive symptoms were the predominant emotional problems before transfer. | Small numbers of participants |
| Korus M, Cruchley E, Stinson JN, Gold A, Anthony SJ. Usability testing of the Internet program: "Teens Taking Charge: Managing My Transplant Online". Pediatr Transplant. 2015 Feb;19(1):107-17. doi: 10.1111/petr.12396. Epub 2014 Dec 12. PMID: 25495484. | Canada | (1) ease of use, (2) efficiency, and (3) acceptability of educational programme. | 21 (8 kidney) | A | Acceptability study | Web-based education tool over period of 3 months. Online modality | Questionnaires | Acceptability scoring | Teens found platform engaging and trustworthy source of information | Convenience sampling and not assessing impact on behaviour. |
| Korus M, Cruchley E, Calic M, Gold A, Anthony SJ, Parekh RS, Stinson JN. Assessing the acceptability and efficacy of teens taking charge: Transplant-A pilot randomized control trial. Pediatr Transplant. 2020 Feb;24(1):e13612. doi: 10.1111/petr.13612. Epub 2019 Nov 19. PMID: 31743564. | Canada | To determine the feasibility and obtain preliminary estimates of efficacy of the online program. | 42 | A | Pilot RCT | 2 year period self-management online resource. Online modality. | Mixed methods- questionnaire quantitative and qualitative | Questionnaire responses | Found it acceptable to use. No difference in health outcomes between intervention and no intervention group | Small study due to pilot nature. |
| Kosola S, Ylinen E, Finne P, Rönnholm K, Ortiz F. Implementation of a transition model to adult care may not be enough to improve results: National study of kidney transplant recipients. Clin Transplant. 2019 Jan;33(1):e13449. doi: 10.1111/ctr.13449. Epub 2018 Dec 12. PMID: 30431669. | Finland | Does a transition model between paediatric and adult care improve graft survival. | 239 | A | Retrospective analysis of case notes. | Group before introduction versus group went through transition clinic. Face-to-face. | Parametric testing | Graft survival | No difference in graft survival between groups. Graft survival lower if transplanted during adolescence vs childhood. | Retrospective study. |
| Kullgren KA, Scholl P, Kidwell KM, Hmiel SP. Using an interactive water bottle to target fluid adherence in pediatric kidney transplant recipients: a pilot study. Pediatr Transplant. 2015 Feb;19(1):35-41. doi: 10.1111/petr.12385. Epub 2014 Nov 12. PMID: 25388882. | USA | To determine whether commercially available interactive water bottle leads to better adherence to fluid intake and subsequent improved graft function. | 32 | A | RCT | Interactive water bottle for 28 days. | Parametric testing | Graft function. Qualitative assessment of content to use. | Overall patients happy to use. More likely to meet fluid targets no change in graft function. | Small study with limited time. Pilot study. |
| McQuillan RF, Toulany A, Kaufman M, Schiff JR. Benefits of a Transfer Clinic in Adolescent and Young Adult Kidney Transplant Patients. *Canadian Journal of Kidney Health and Disease*. January 2015. doi:[10.1186/s40697-015-0081-6](https://doi.org/10.1186/s40697-015-0081-6) | Canada | To investigate whether a kidney transplant transfer clinic for adolescent and young adult kidney transplant recipients transitioning from pediatric to adult care improves adherence post-transfer | 32 | YA | Retrospective cohort study | Joint kidney transplant transfer clinic between a pediatric kidney transplant program, adult kidney transplant program, and adolescent medicine at two academic health centres. Face-to-face. | Chi-square or Fisher’s exact test | Non-adherence was defined as either self-reported medication nonadherence or displaying two of the following three characteristics: non-attendance at clinic, non-attendance for blood work appointments, or undetectable calcineurin inhibitor levels within 1 year post-transfer | Attendance at a single kidney transplant transfer clinic was associated with improved adherence and renal function in the year following transfer to adult care | Non-detectable tacrolimus levels- did not account for lower than desired levels. Self reported. |
| Michaud V, Achille M, Chainey F, Phan V, Girardin C, Clermont MJ. Mixed-methods evaluation of a transition and young adult clinic for kidney transplant recipients. Pediatr Transplant. 2019 Jun;23(4):e13450. doi: 10.1111/petr.13450. Epub 2019 May 7. PMID: 31062926. | Canada | To describe the experiences of kidney transplant patients attending a young adult clinic or a regular adult clinic | 33 | YA | Mixed method- quantitative and qualitative | Comparison between those attending a standard clinic and a specialised transition clinic. Face-to-face. | Thematic analysis for qualitative data. Two sample T-Test for quantitative data. | Health Care Climate Questionnaire. Treatment Self‐ Regulation Questionnaire. Perceived Competence Scale. Physical Component Summary (PCS‐12) and Mental Component Summary (MCS‐12).KTQ‐25: Kidney Transplant Questionnaire‐25. | Four themes characterized patients' experiences: resilience; relational needs and the therapeutic alliance; quest for balance; and quest for normalcy. clinically significant differences on tacrolimus blood levels variability, self-reported adherence, and physical quality of life | Small sample size, retrospective analysis. French versions of questionnaires not validated. |
| Nguyen C, Dew MA, Irizarry T, McNulty M, Rennick J, Knäuper B, Descoteaux A, Grenier A, Jeannot L, Foster BJ, DeVito Dabbs AJ; all TAKE-IT TOO Investigators. Promoting medication adherence from the perspective of adolescent and young adult kidney transplant recipients, parents, and health care professionals: A TAKE-IT TOO study. Pediatr Transplant. 2020 Aug;24(5):e13709. doi: 10.1111/petr.13709. Epub 2020 May 10. PMID: 32388916; PMCID: PMC7392786. | Canada/ US | Following on from previous trial to involve stakeholders in design using qualitative methodology for interventions to improve adherence. | 32 | Both | Qualitative | User engagement in design, feasibility and practicality of interventions in real world | Content analysis | Questionnaires and focus groups | The stakeholders also made suggestions for the multi-component behavioral intervention, including an expanded electronic pillbox and companion website, education materials, and customized digitized features to support shared responsibility and communication among recipients, parents, and health professionals. Several suggestions regarding the functionality and features of the potential intervention reported in this early stage will be explored in more depth as the iterative process unfolds | Potential selection bias with regards to those willing to take part. |
| Pai AL, Ingerski LM, Perazzo L, Ramey C, Bonner M, Goebel J. Preparing for transition? The allocation of oral medication regimen tasks in adolescents with renal transplants. Pediatr Transplant. 2011 Feb;15(1):9-16. doi: 10.1111/j.1399-3046.2010.01369.x. Epub 2010 Sep 30. PMID: 20880382. | USA | To examine strategies that families use to manage the post-transplant oral medication regimen of adolescents with renal transplants. | 17 | A | Mixed method. | Semi-structured interviews. Family derived solutions. Adherence was assessed via electronic pill bottles and calculated by dividing the number of doses taken by those prescribed | Qualitative analysis- thematic analysis. Bivariate correlation. | Semi-structured interviews. MEMS bottle measuring pills. | Patients and their caregivers identified the following tasks as part of the oral medication regimen: filling the pillbox (71%), calling for refills (65%) and verifying that the pillbox is filled correctly (47%). Adult caregivers were primarily responsible for managing the medication regimen for the majority of adolescents (70%). Secondary analyses revealed that the number of oral medication regimen tasks identified by the dyad was significantly associated with patient adherence. | Not all participant data was audio recorded and coded but nots taken. Small study hard to generalise. |
| Pape L, Lämmermühle J, Oldhafer M, Blume C, Weiss R, Ahlenstiel T. Different models of transition to adult care after pediatric kidney transplantation: a comparative study. Pediatr Transplant. 2013 Sep;17(6):518-24. doi: 10.1111/petr.12102. Epub 2013 Jun 4. PMID: 23730905. | Germany | Whether a specialised transition clinic, a transplant clinic or a clinic with general nephrologist affected clinical outcomes such as graft loss, medication changes and patient satisfaction | 66 | A | Retrospective observational study | Clinical data reviewed from medical records. Questionnaires sent to participants to assess. Face-to-face. | Descriptive statistical analyses | Clinical outcomes | Three patients developed graft loss. GFR development was comparable in the three settings. Immunosuppressive therapy was stable in setting 1, whereas the number of changes increased in setting 2 and even more in setting 3. The percentage of patients with steroids increased from 36% to 38% and 52% in settings 1–3. Patient satisfaction was highest in setting 1 (100% vs. 64% and 78%, p < 0.05). Setting 1 was associated with fewer changes in therapy (13% vs. 91% and 45%, p < 0.05). | Third of patients lost to follow-up. Questionnaire not taken at time of transition but retrospectively. Small number of patients. |
| Pollack AH, Snyder J. Reflecting on patient-generated photographs of the pediatric renal transplant experience. Pediatr Transplant. 2020 Oct 28:e13896. doi: 10.1111/petr.13896. Epub ahead of print. PMID: 33111458. | USA | Photograph-elicitation, a qualitative method where images were used to prompt individuals to talk about their personal experiences and values | 24 | Both | Qualitative study | Semi-structured interviews with prompting using photos to stimulate reflection. | Thematic analysis. | Discussion associated with photographs. | a) sensemaking; (b) transitions and agency; (c) social interactions and community engagement; and (d) barriers and obstacles. | Small study. Outcomes not linked to graft function. |
| Prestidge C, Romann A, Djurdjev O, Matsuda-Abedini M. Utility and cost of a renal transplant transition clinic. Pediatr Nephrol. 2012 Feb;27(2):295-302. doi: 10.1007/s00467-011-1980-0. Epub 2011 Aug 9. PMID: 21823039. | Canada | Determine if a transition clinic improved outcomes and whether it was a cost effective intervention | 33 | A | Cost-effectiveness study | Transition clinic- dedicated clinic, closer follow-up, multidisciplinary team. Face-to-face. | Death and allograft loss measured as outcomes. Costs calculated. | Mortality and allograft loss. | Transition clinic associated with reduced mortality and improved allograft survival | Vocation outcomes, quality of life and other important aspects were not measured in this study |
| Szpotanska-Sikorska M, Pietrzak B, Wielgos M. Contraceptive awareness and birth control selection in female kidney and liver transplant recipients. Contraception. 2014 Oct;90(4):435-9. doi: 10.1016/j.contraception.2014.04.014. Epub 2014 May 4. PMID: 24909634. | Poland | to assess birth control selection and the role of contraceptive counselling sessions in female transplant recipients. | 137 | Both | Questionnaire pre and post education sessions | Normal transplant education- face-to-face. | Knowledge of contraceptive methods | Knowledge identified in questionnaire | The selection of effective contraception in KT recipients remain suboptimal. Despite the low numbers of women who received contraceptive counselling in this study, consultation was nonetheless associated with choosing an effective method of contraception. | Single centre study |
| Wallace J, Yorgin PD, Carolan R, Moore H, Sanchez J, Belson A, Yorgin L, Major C, Granucci L, Alexander S, Arrington D. The use of art therapy to detect depression and post-traumatic stress disorder in pediatric and young adult renal transplant recipients. Pediatr Transplant. 2004 Feb;8(1):52-9. doi: 10.1046/j.1397-3142.2003.00124.x. PMID: 15009841. | USA | (1) determine the prevalence of depression and emotional trauma and (2) assess the utility of the Formal Elements of Art Therapy Scale (FEATS) | 64 | Both | Non‐randomized cross‐sectional cohort analysis | Art based therapy. | Using self‐report measures (CDI and Davidson) and art‐based assessments. Subject art was analyzed by art therapists using seven of the 14 elements of the (FEATS), to assess depression | Detection and treatment of depression. | Suggest that while art therapy may be of utility in the identification of pediatric and young adult transplant recipients who are suffering from depression, FEATS analysis appears to lack sufficient sensitivity to warrant its use in this population | Not-blinded. Pateints selection aimed at more recent recipients and patients who were identified as having problems. |
| Weigmann-Faßbender S, Pfeil K, Betz T, Sander A, Weiß K, Tönshoff B, Friedmann-Bette B. Physical fitness and health-related quality of life in pediatric renal transplant recipients: An interventional trial with active video gaming. Pediatr Transplant. 2020 Feb;24(1):e13630. doi: 10.1111/petr.13630. Epub 2019 Dec 27. PMID: 31880043. | Germany | If active videogaming would improve cardiovascular fitness in transplant recipients | 22 | A | Interventional control observational study | Spiroergometry, a motor coordination test, and a maximal handgrip strength test. Quality of life was determined with a validated questionnaire, and daily physical activity was recorded with a physical activity monitor | Comparison pre and post intervention | Improvement in quality of life, logged exercise activity and fitness measures. | The renal transplant recipients exhibited a substantial impairment compared with the controls in peak oxygen consumption (−31%, *P* < .001), motor competence (−44%, *P* < .001), daily physical activity (−33%, *P* = .001), and quality of life (−12%, *P* = .017). Handgrip strength was similar in both groups. Despite of low compliance in the intervention group, steps per hour were significantly increased after 6 weeks of exergaming (+31%, *P* = .043); however, all other measures remained unchanged. | low number of investigated patients and the inter-individual variability with regard to their medication with potential (small) impact on cardiorespiratory performance capacity, for example, beta blockers |
| Weitz M, Heeringa S, Neuhaus TJ, Fehr T, Laube GF. Standardized multilevel transition program: Does it affect renal transplant outcome? Pediatr Transplant. 2015 Nov;19(7):691-7. doi: 10.1111/petr.12570. Epub 2015 Aug 11. PMID: 26260514. | Switzerland | To determine the effects of the transition program on eGFR and number of ARs in comparison to a group without a transition program at one and three yr after transfer | 59 (33 in transition clinic) | A | single‐center retrospective cohort study | Structured multidisciplinary transition clinic. Face-to-face clinic. | Review of clinical notes and tests. | Acute rejection episodes and eGFR. Multiple regression analysis. | Multiple regression analysis revealed a significantly lower decline of eGFR in the group with transition program (−11.3 ± 44 mL/min/1.73 m^2^) compared to the group without transition program (−28.4 ± 33 mL/min/1.73 m^2^) at three yr after transfer. The number of AR episodes significantly decreased from 34.6% in the group without transition program to 9.1% in the group with transition program. | Small sample size. Retrospective. Possible confounders like HLA matching not part of analysis. |
